# Simplified Outcome Prediction in Patients Undergoing Transcatheter Tricuspid Valve Intervention by Survival Tree-Based Modelling

**DOI:** 10.1016/j.jacadv.2024.101575

**Published:** 2025-01-22

**Authors:** Vera Fortmeier, Mark Lachmann, Lukas Stolz, Jennifer von Stein, Karl-Philipp Rommel, Mohammad Kassar, Muhammed Gerçek, Anne R. Schöber, Thomas J. Stocker, Hazem Omran, Michelle Fett, Jule Tervooren, Maria I. Körber, Amelie Hesse, Gerhard Harmsen, Kai Peter Friedrichs, Shinsuke Yuasa, Tanja K. Rudolph, Michael Joner, Roman Pfister, Stephan Baldus, Karl-Ludwig Laugwitz, Stephan Windecker, Fabien Praz, Philipp Lurz, Jörg Hausleiter, Volker Rudolph

**Affiliations:** a Department of General and Interventional Cardiology, Heart and Diabetes Center North Rhine-Westphalia, Ruhr University Bochum, Bad Oeynhausen, Germany; b First Department of Medicine, Klinikum rechts der Isar, Technical University of Munich, Munich, Germany; c DZHK (German Center for Cardiovascular Research), Partner Site Munich Heart Alliance, Munich, Germany; d Medizinische Klinik und Poliklinik I, Klinikum der Universität München, Ludwig Maximilians University of Munich, Munich, Germany; e Department of Cardiology, Heart Center, University of Cologne, Cologne, Germany; f Department of Cardiology, Heart Center Leipzig, University of Leipzig, Leipzig, Germany; g Department of Cardiology, Inselspital Bern, Bern University Hospital, Bern, Switzerland; h Department of Physics, University of Johannesburg, Auckland Park, South Africa; i Department of Cardiovascular Medicine, Okayama University, Okayama, Japan; j Department of Cardiology, German Heart Center Munich, Technical University of Munich, Munich, Germany

**Keywords:** machine learning, transcatheter tricuspid valve intervention, tricuspid regurgitation

## Abstract

**Background:**

Patients with severe tricuspid regurgitation (TR) typically present with heterogeneity in the extent of cardiac dysfunction and extra-cardiac comorbidities, which play a decisive role for survival after transcatheter tricuspid valve intervention (TTVI).

**Objectives:**

This aim of this study was to create a survival tree-based model to determine the cardiac and extra-cardiac features associated with 2-year survival after TTVI.

**Methods:**

The study included 918 patients (derivation set, n = 631; validation set, n = 287) undergoing TTVI for severe TR. Supervised machine learning-derived survival tree-based modelling was applied to preprocedural clinical, laboratory, echocardiographic, and hemodynamic data.

**Results:**

Following univariate regression analysis to pre-select candidate variables for 2-year mortality prediction, a survival tree-based model was constructed using 4 key parameters. Three distinct cluster-related risk categories were identified, which differed significantly in survival after TTVI. Patients from the low-risk category (n = 261) were defined by mean pulmonary artery pressure ≤28 mm Hg and N-terminal pro–B-type natriuretic peptide ≤2,728 pg/mL, and they exhibited a 2-year survival rate of 85.5%. Patients from the high-risk category (n = 190) were defined by mean pulmonary artery pressure >28 mm Hg, right atrial area >32.5 cm^2^, and estimated glomerular filtration rate ≤51 mL/min, and they showed a significantly worse 2-year survival of only 52.6% (HR for 2-year mortality: 4.3, *P* < 0.001). Net re-classification improvement analysis demonstrated that this model was comparable to the TRI-Score and outperformed the EuroScore II in identifying high-risk patients. The prognostic value of risk phenotypes was confirmed by external validation.

**Conclusions:**

This simple survival tree-based model effectively stratifies patients with severe TR into distinct risk categories, demonstrating significant differences in 2-year survival after TTVI.

A graded relationship between severity of tricuspid regurgitation (TR) and risk of mortality has been shown across a broad range of patient populations irrespective of left heart disease, pulmonary hypertension, and right ventricular dysfunction.^1^ Patients with severe TR often face a high surgical risk, leading to a large number not receiving treatment beyond conservative management.^2^ Transcatheter tricuspid valve interventions (TTVIs) including leaflet approximation, direct annuloplasty, and valve implantation (both orthotopic and heterotopic) have meanwhile emerged as a safe treatment alternative, fueling the hope to reduce the need for heart failure hospitalizations and to prolong survival in an otherwise multimorbid and eventually moribund patient collective.^3^ Notably, the distressing mortality rate of ∼10% in patients undergoing isolated tricuspid valve surgery can be at least partly attributed to delayed referrals and advanced disease states. These circumstances often result in extra-cardiac damage to vital end-organs, such as the liver and kidney, further worsening the prognosis in these patients.^4^

Considering the prognostic impact of both cardiac and extra-cardiac damage, while also acknowledging the heterogeneity in the underlying pathophysiology, disease progression, and prevalence of comorbidities typically encountered in patients with severe TR undergoing TTVI, this study sought to employ a supervised machine learning technique to unveil pathophysiologically and prognostically relevant risk phenotypes. Machine learning algorithms, distinct from hypothesis-driven approaches, derive insights directly from the data itself.^5^^,^^6^ Therefore, the development of a survival tree-based model appears as particularly advantageous as it does not infer causality between severe TR and accumulated (extracardiac) pathologies, nor does it presuppose a strict linear sequence in the occurrence of severe TR, extra-cardiac damage, and mortality. The underlying concept of a survival tree-based model is to perform a hierarchically organized risk stratification with binary decision nodes at each level ultimately allocating the patient to distinct risk phenotypes.^7^^,^^8^ As such, survival tree-based models are easy to comprehend and similarly easy to communicate to the patient, and by balancing the power of machine learning with an easy-to-apply risk-assessment tool, they are well-suited to be integrated into clinical decision-making.

Therefore, the aim of this study was to use survival tree-based modelling to determine the cardiac and extra-cardiac factors associated with 2-year survival after TTVI. The secondary aims were to: 1) compare the survival tree-based model to identifying high-risk patients with the TRI-Score and the EuroScore II; and 2) to examine the impact of residual TR on survival prognostication after TTVI.

## Methods

### Study population and data collection

This is a post hoc, multicentric analysis (5 high-volume centers in Germany and Switzerland) of prospectively and systematically collected data from 918 patients undergoing isolated TTVI for severe TR between 2016 and 2022.

The key inclusion criterion focused on patients with severe TR and a high symptomatic burden or who were deemed inoperable due to prohibitive perioperative risk as assessed by the local heart team. Etiologies of TR included primary TR, secondary TR (subdivided into functional atrial and ventricular TR), and TR related to cardiac implantable electronic devices.

Planned and conducted in conformity with the Declaration of Helsinki, the study was approved by the local ethics committee of each center, and all patients gave their written informed consent.

#### Clinical endpoint

Two-year all-cause mortality was the primary outcome of interest.

#### Statistical analysis

Survival was illustrated using the Kaplan-Meier method, and the log-rank test was applied to compare survival rates. Moreover, a Cox proportional hazards model was used to estimate HRs.

Before developing the survival tree-based model through supervised recursive partitioning within the derivation cohort, a univariate Cox regression analysis on a complementary set of clinical, laboratory, echocardiographic, and hemodynamic variables was performed to identify factors related to all-cause mortality. Variables with a *P* ≤ 0.05 in univariate testing were subsequently considered as input parameters for the survival tree-based model.

Following imputation, the construction of the survival tree-based model was performed through 5-fold cross-validation using the “partykit” R package. Each fold of the data was used once as a validation while the k-1 remaining folds formed the training set. Furthermore, we restricted the survival tree-based model to a maximum of 3 decision nodes to prevent overfitting and to ensure sufficient sample sizes within each cluster. The fitted survival tree-based model was hereinafter applied to patient data from the external validation cohort, and 1- and 2-year survival rates per cluster were then compared between derivation and validation cohorts to test the predictive validity of the model.

Net reclassification analysis compares the survival tree-based model to the TRI-Score and the EuroScore II in identifying high-risk patients.

A *P* value ≤0.05 was conside red to indicate statistical significance. All statistical analyses were performed using R statistical software (R version 4.3.2, R Foundation for Statistical Computing) (Supplemental Table 1 for a complete list of employed R packages).

A more detailed description of methods is included in the Supplemental Material.

## Results

### Baseline characteristics

Data from a total of 918 patients (median age: 80.0 years; IQR: 75.7-83.1 years) undergoing TTVI for severe TR were used for this analysis. Among them, there were 405 (44.1%) men. Patients typically presented with dyspnea corresponding to NYHA functional class III (76.8%) or IV (13.2%), and with a median N-terminal pro–B-type natriuretic peptide (NT-proBNP) level of 2,304 (IQR: 1,336-4,634) pg/mL (Table 1). Severe, massive, and torrential TR were diagnosed in 475 (51.7%), 288 (31.4%), and 155 (16.9%) patients, respectively, (Table 2). Moreover, 830 patients (90.4%) suffered from atrial fibrillation, and median mean pulmonary artery pressure (mPAP) levels were elevated to 29 (IQR: 24-36) mm Hg. Renal function expressed as median estimated glomerular filtration rate (eGFR) levels ranged at 47 (IQR: 33-64) mL/min.

**Table 1:** Demographic and Clinical Baseline Characteristics of the Study Population

	All Patients (N = 918)	Derivation Cohort (n = 631)	Validation Cohort (n = 287)	*P* Value
Age, y	80.0 (75.7-83.1)	79.9 (75.9-83.0)	80.3 (75.0-83.2)	0.738
Men	405 (44.1%)	302 (47.9%)	103 (35.9%)	<0.001
BMI, kg/m^2^	25.3 (22.7-28.7)	25.2 (22.7-28.7)	25.4 (22.5-28.7)	0.731
Arterial hypertension	769 (83.8%)	534 (84.6%)	235 (81.9%)	0.342
Diabetes mellitus	241 (26.3%)	170 (26.9%)	71 (24.7%)	0.534
NYHA functional class ≤ II	92 (10.0%)	47 (7.45%)	45 (15.7%)	<0.001
NYHA functional class III	705 (76.8%)	505 (80.0%)	200 (69.7%)	<0.001
NYHA functional class IV	121 (13.2%)	79 (12.5%)	42 (14.6%)	0.440
EuroScore II, %	4.80 (2.96-8.24)	4.76 (2.86-7.80)	4.80 (3.16-8.90)	0.230
TRI-Score, points	5 (4-7)	6 (4-7)	5 (3-6)	0.001
eGFR, mL/min	47 (33-64)	52 (36-67)	40 (29-54)	<0.001
NT-proBNP, pg/mL	2,304 (1,336-4,634)	2,402 (1,326-4,935)	2,183 (1,342-4,203)	0.420
Hemoglobin, g/dL	11.2 (9.3-12.9)	11.0 (8.8-12.7)	11.7 (10.4-13.1)	<0.001
Bilirubin, mg/dL	0.82 (0.58-1.18)	0.90 (0.60-1.20)	0.70 (0.50-1.09)	0.001
AST, U/L	28 (23-36)	29 (24-36)	28 (23-34)	0.562
ALT, U/L	19 (14-25)	19 (14-25)	18 (14-23)	0.500
gGT, U/L	97 (55-185)	97 (54-186)	108 (57-163)	0.932
CAD	386 (42.0%)	270 (42.8%)	116 (40.4%)	0.547
COPD	167 (18.2%)	112 (17.7%)	55 (19.2%)	0.673
Atrial fibrillation	830 (90.4%)	560 (88.7%)	270 (94.1%)	0.015
Pacemaker	253 (27.6%)	184 (29.2%)	69 (24.0%)	0.126
TR etiology				
Ventricular	503 (54.8%)	411 (65.1%)	92 (32.1%)	<0.001
Atrial	325 (35.4%)	155 (24.6%)	170 (59.2%)	<0.001
CIED-related	57 (6.21%)	38 (6.02%)	19 (6.62%)	0.841
Primary	33 (3.59%)	27 (4.28%)	6 (2.09%)	0.144

Values are median (IQR) or n (%).

ALT = alanine aminotransferase; AST = aspartate aminotransferase; BMI = body mass index; CAD = coronary artery disease; COPD = chronic obstructive pulmonary disease; eGFR = estimated glomerular filtration rate; gGT = gamma-glutamyl transferase.

**Table 2:** Echocardiographic and Hemodynamic Baseline Characteristics of the Study Population

	All Patients (N = 918)	Derivation Cohort (n = 631)	Validation Cohort (n = 287)	*P* Value
LVEF, %	55 (49-61)	55 (49-62)	55 (50-60)	0.928
LVESD, mm	35 (29-46)	37 (29-48)	32 (27-39)	<0.001
LVEDD, mm	47 (42-53)	48 (43-53)	47 (42-52)	0.050
LA volume, mL	83 (49-118)	107 (82-1,151)	59 (40-88)	<0.001
sPAP_echocardiography_, mm Hg	40 (31-50)	38 (29-48)	43 (35-53)	<0.001
TAPSE, mm	17 (14-20)	17 (14-20)	17 (14-20)	0.125
RV FAC, %	39 (32-46)	41 (33-48)	35 (30-40)	<0.001
Basal RV diameter, mm	46 (42-52)	46 (41-52)	46 (42-52)	0.742
TV EROA, cm^2^	0.54 (0.40-0.78)	0.50 (0.40-0.72)	0.58 (0.43-0.90)	0.003
TV regurgitation volume, mL	45 (35-61)	45 (34-61)	45 (36-61)	0.653
TR vena contracta width, mm	10 (8-14)	10 (8-13)	11 (8-14)	0.019
TR ≤ III/V°	475 (51.7%)	347 (55.0%)	128 (44.6%)	0.004
TR = IV/V°	288 (31.4%)	197 (31.2%)	91 (31.7%)	0.944
TR = V/V°	155 (16.9%)	87 (13.8%)	68 (23.7%)	<0.001
RA area, cm^2^	36 (29-45)	35 (28-43)	38 (30-52)	<0.001
Inferior vena cava diameter, mm	25 (21-29)	25 (21-30)	25 (21-28)	0.170
sPAP_invasive_, mm Hg	45 (37-55)	45 (37-56)	44 (37-52)	0.187
dPAP, mm Hg	19 (14-24)	19 (14-24)	18 (14-23)	0.182
mPAP, mm Hg	29 (24-36)	29 (24-36)	29 (24-35)	0.828
mPCWP, mm Hg	19 (14-24)	19 (14-25)	19 (15-23)	0.552
Concomitant AS ≥ II/III°	40 (4.36%)	26 (4.12%)	14 (4.88%)	0.729
Concomitant MR ≥ II/IV°	230 (25.1%)	122 (19.3%)	108 (37.6%)	<0.001

Values are median (IQR) or n (%).

AS = aortic stenosis; dPAP = diastolic pulmonary artery pressure (as assessed by right-heart catheterization); LA = left atrial; LVEDD = left ventricular end-diastolic diameter; LVEF = left ventricular ejection fraction; LVESD = left ventricular end-systolic diameter; mPAP = mean pulmonary artery pressure (as assessed by right-heart catheterization); mPCWP = mean postcapillary wedge pressure (as assessed by right-heart catheterization); MR = mitral regurgitation; RA = right atrial; RV = right ventricular; RV FAC = right ventricular fractional area change; sPAP_echocardiography_ = systolic pulmonary artery pressure (as assessed by echocardiography); sPAP_invasive_ = systolic pulmonary artery pressure (as assessed by right-heart catheterization); TAPSE = tricuspid annular plane systolic excursion; TR = tricuspid regurgitation; TV = tricuspid valve; TV EROA = tricuspid valve effective regurgitant orifice area.

**Figure 1:**
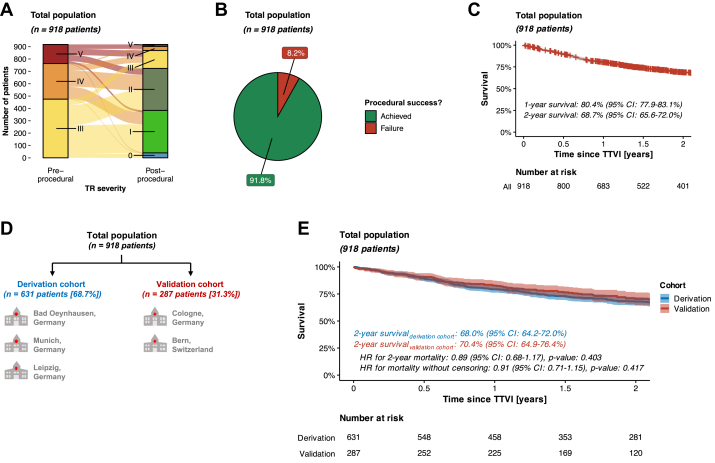
**General Information About the Study Population** (A) Alluvial diagram comparing pre- and post-procedural TR severity. (B) Pie chart comparing rates of procedural success (see Methods section for the definition of procedural success). (C) Kaplan-Meier survival plot for the entire study population (restricted to 2 years after TTVI). (D) Study scheme to train and validate the survival tree-based model to predict mortality in patients undergoing TTVI. Most importantly, the external validation cohort comprised 287 patients from 2 independent institutions that have not been involved in model development. Thus, the external validation may render a true estimate of how well the algorithm would perform in future (ie, never-before-seen) patients. (E) Kaplan-Meier survival analysis comparing survival between derivation and external validation cohorts. TR = tricuspid regurgitation; TTVI = transcatheter tricuspid valve intervention.

Transcatheter edge-to-edge repair was the predominant TTVI technique being performed in 749 (81.6%) patients. This was followed by annuloplasty in 162 (17.6%) patients, and valve implantation in 7 (0.8%) patients. A successful TR reduction by at least one grade could be achieved in 843 (91.8%) out of 918 cases (Table 3, Figures 1A and 1B). While 341 deaths among 918 enrolled patients were recorded, survivors were traced on a median follow-up time of 2.19 years (IQR: 1.40-3.63 years) (Supplemental Figure 2). Accordingly, 1- and 2-year survival rates for all patients ranged at 80.4% and 68.7%, respectively (Figure 1C).

**Table 3:** Procedural Characteristics and Postprocedural Outcomes per Cohort

	All Patients (N = 918)	Derivation Cohort (n = 631)	Validation Cohort (n = 287)	*P* Value
History of cardiac surgery	240 (26.1%)	163 (25.8%)	77 (26.8%)	0.812
Prior tricuspid valve surgery	9 (0.98%)	9 (1.43%)	0 (0%)	0.095
Prior TTVI	21 (2.29%)	14 (2.22%)	7 (2.44%)	1.00
Procedural time, min	120 (84-173)	103 (75-150)	168 (130-212)	<0.001
TR reduction by at least one grade	843 (91.8%)	599 (94.9%)	244 (85.0%)	<0.001
Residual TR ≤ II/V°	724 (78.9%)	536 (84.9%)	188 (65.5%)	<0.001

Values are n (%) or median (IQR).

TR = tricuspid regurgitation; TTVI = transcatheter tricuspid valve intervention.

To develop and validate a model for categorizing cardiac and extra-cardiac damage in patients with severe TR, we divided the study population into 2 groups: a derivation cohort, comprising patients from 3 hospitals, and an external validation cohort, consisting of patients from 2 independent hospitals (Figure 1D). Notably, the cohorts exhibited broadly comparable clinical, echocardiographic, and hemodynamic profiles (Tables 1 and 2). Moreover, survival analysis following TTVI revealed no significant differences between the derivation and validation cohorts (Figure 1E), indicating a consistent treatment effect across different hospital settings.

### Development of the survival tree-based model

To identify factors being associated with mortality after TTVI, a univariate Cox regression analysis was conducted within the derivation cohort. This analysis identified 16 significant predictors of mortality from a comprehensive set of clinical, laboratory, echocardiographic, and hemodynamic variables (Table 4). These predictors contributed in the construction of a survival tree-based model for risk stratification. Before starting model development, however, missing values among those 16 parameters had to be imputed (Supplemental Figure 3). In total, there were 479 (4.74%) out of 10,096 data points missing, with the largest proportion of missing values being measurements of left ventricular end-diastolic diameter (26.1%).

**Table 4:** Univariate Cox Regression Analysis With Uncensored Mortality After TTVI as a Dependent Variable (Derivation Cohort)

	Univariate Analysis	
	HR (95% CI)	*P* Value
Age (increment per 10 y)	1.04 (0.88-1.23)	0.623
Male	1.40 (1.09-1.80)	0.009
Diabetes mellitus	1.40 (1.07-1.82)	0.014
Arterial hypertension	0.96 (0.68-1.35)	0.791
CAD	1.42 (1.10-1.82)	0.006
COPD	1.18 (0.86-1.61)	0.300
Atrial fibrillation	0.80 (0.56-1.15)	0.222
eGFR (increment per 10 mL/min)	0.85 (0.80-0.91)	<0.001
NT-proBNP (increment per 2,000 pg/mL)	1.06 (1.04-1.08)	<0.001
Hemoglobin (increment per 1 g/dL)	0.91 (0.86-0.95)	<0.001
Bilirubin (increment per 1 mg/dL)	0.99 (0.92-1.07)	0.781
AST (increment per 10 U/L)	1.03 (0.95-1.11)	0.475
ALT (increment per 10 U/L)	0.80 (0.69-0.92)	0.002
gGT (increment per 10 U/L)	1.01 (1.00-1.02)	0.002
NYHA class (increment per class)	1.46 (1.13-1.90)	0.004
LVEF (increment per 1%)	0.96 (0.97-1.00)	0.006
LVEDD (increment per 10 mm)	1.23 (1.02-1.49)	0.030
LA volume (increment per 10 mL)	1.03 (0.99-1.06)	0.181
mPAP (increment per 10 mm Hg)	1.45 (1.28-1.65)	<0.001
TAPSE (increment per 10 mm)	0.95 (0.92-0.97)	<0.001
Basal RV diameter (increment per 10 mm)	1.12 (0.96-1.31)	0.136
TV regurgitation volume (increment per 10 mL)	1.02 (0.97-1.08)	0.491
TV EROA (increment per 1 cm^2^)	1.48 (1.03-2.13)	0.034
TR vena contracta width (increment per 1 mm)	1.04 (1.01-1.07)	0.004
RA area (increment per 10 cm^2^)	1.15 (1.03-1.28)	0.010

Abbreviations as in Tables 1 and 2.

The survival tree-based model employed recursive partitioning, thus establishing 4 key decision nodes—mPAP, NT-proBNP, right atrial (RA) area, and eGFR—to stratify patients into 5 distinct clusters (Central Illustration). Such a machine learning-guided selection of parameters facilitated a comprehensive evaluation of:

• hemodynamic status in terms of pulmonary hypertension (indicated by mPAP levels),
• structural alterations of the right heart expressed as RA enlargement,
• severity of decompensated heart failure expressed as NT-proBNP levels, and
• extra-cardiac organ impairment expressed as renal dysfunction (reflected by eGFR measurements).

**Central Illustration:**
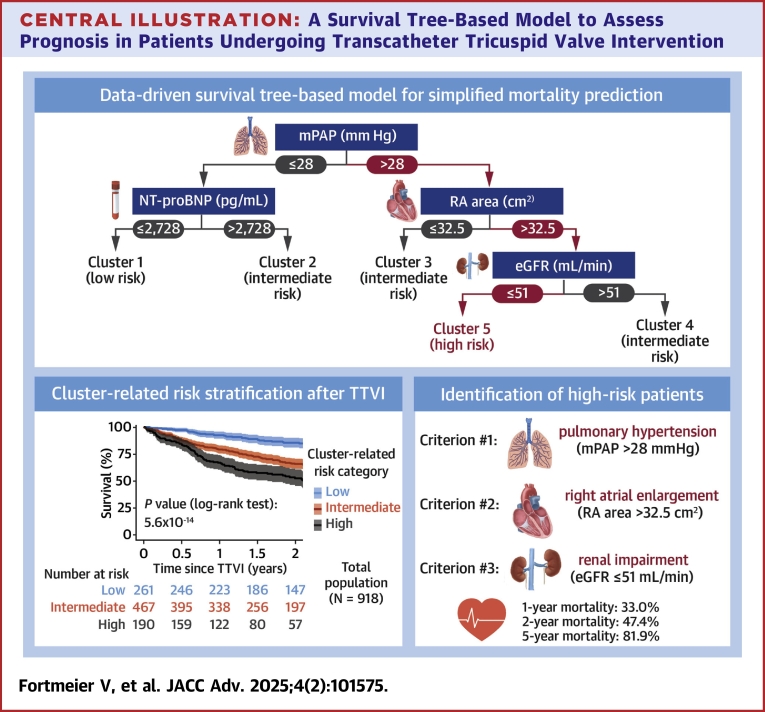
**A Survival Tree-Based Model to Assess Prognosis in Patients Undergoing Transcatheter Tricuspid Valve Intervention** A machine learning-derived survival tree-based model established on a complementary set of laboratory, echocardiographic, and hemodynamic parameters facilitates to capture the complexity of both cardiac and extra-cardiac impairments as commonly encountered in patients presenting with severe tricuspid regurgitation, and it aids in assessing prognosis after the transcatheter tricuspid valve intervention by assigning patients to cluster-related risk categories.

**Figure 2:**
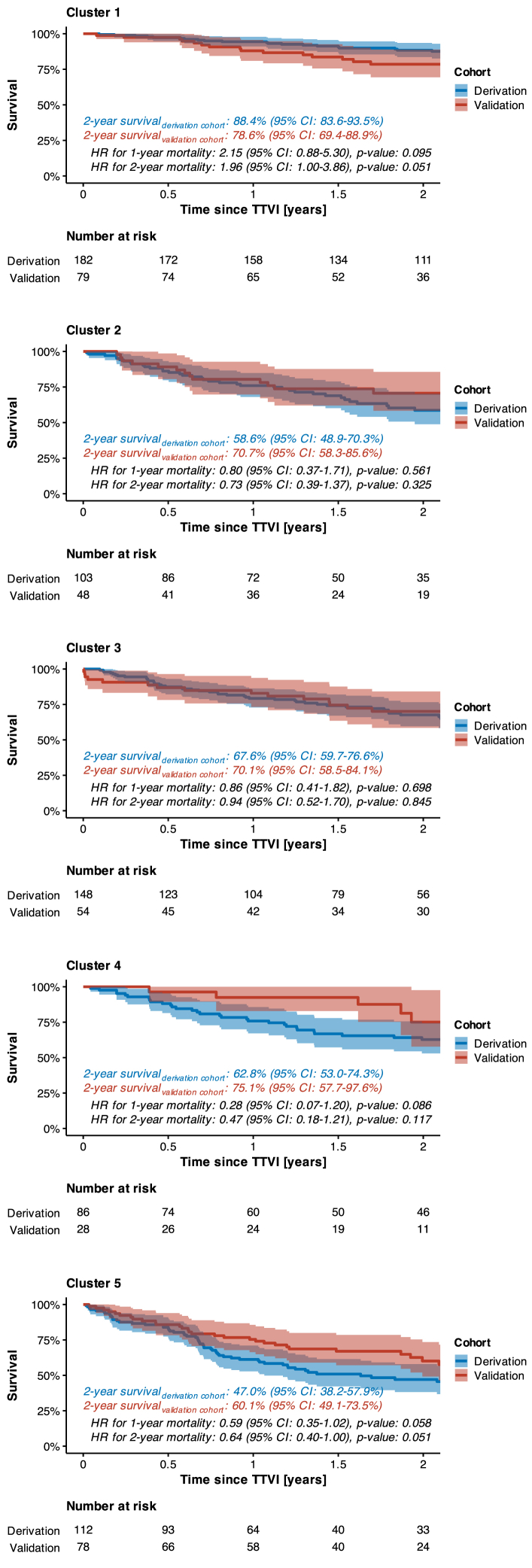
Comparison of 2-Year Survival Rates With Survival Tree-Based Modelling (Considering Derivation and Validation Cohorts Separately)

Importantly, the survival rates after TTVI differed significantly across clusters (Central Illustration); for instance, Cluster 1 (characterized by mPAP ≤28 mm Hg and NT-proBNP ≤2,728 pg/mL) exhibited a 2-year survival rate of 88.4%, which was significantly higher than the 2-year survival rate of 47% observed in Cluster 5 (defined by mPAP >28 mm Hg, RA area >32.5 cm^2^, and eGFR ≤51 mL/min). Furthermore, the robustness of the survival tree-based model was validated externally with a cohort of 287 patients from 2 independent hospitals, confirming its predictive validity for 2-year survival after TTVI (Figure 2, Supplemental Figure 4). Beyond the primary study endpoint of 2-year all-cause mortality, the survival tree-based model was also effective in stratifying patients for 5-year survival after TTVI, ranging from 64.8% in Cluster 1 to as low as 18.1% in Cluster 5 (Supplemental Figure 5). It is noteworthy, however, that the overall number of patients at risk at 5 years after TTVI was relatively small (only 51 patients) (Supplemental Figure 2C). Despite this limitation, the perspective on 5-year survival after TTVI is still valuable, as it ensures that our model provides insights not just in the short term but also in the long term, confirming that survival curves do not converge over time but continue to provide distinct prognostic stratification.

### Characterization of cluster-related risk categories

According to survival rates after TTVI, clusters were then aggregated to risk categories, meaning that Cluster 1 was classified as low-risk, Clusters 2-4 as intermediate-risk, and Cluster 5 as high-risk category (Central Illustration).

**Figure 3:**
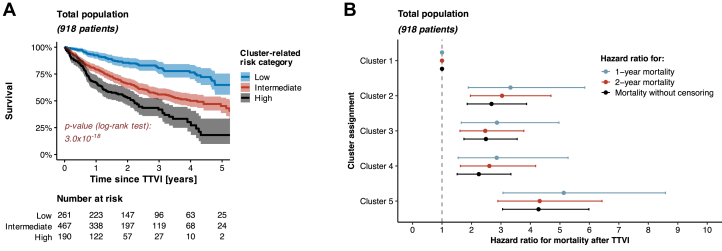
**Survival Beyond the Primary Study Endpoint of 2 Years After TTVI** (A) Kaplan-Meier survival analysis per cluster-related risk category (restricted to 5 years after TTVI). (B) Forest plot to illustrate the HR for mortality in accordance with cluster assignment (total population).

Patients from the low-risk category (261 patients) presented with lowest median NT-proBNP levels (1,347 [IQR: 818-1,848] pg/mL) and a comparatively lower prevalence of severe dyspnea corresponding to NYHA functional class IV (8.81%) (Table 5). Moreover, these patients exhibited regular left-heart function (median left ventricular ejection fraction: 58% [IQR: 53%-63%]), the least-elevated mPAP levels (median: 23 [IQR: 20-26] mm Hg), and a preserved right ventricular function (median tricuspid annular plane systolic excursion [TAPSE]: 18 [IQR: 15-21] mm) (Table 6). Renal function was also the best among all risk categories (median eGFR: 57 [IQR: 43-72] mL/min). Notably, the proportion of patients being diagnosed with an atrial form of secondary TR was highest among patients from the low-risk category (40.2%). Accordingly, 1-year, 2-year, and 5-year survival rates after TTVI in patients from low-risk category ranged at 92.5%, 85.5%, and 64.8%, respectively (Central Illustration, Figure 3).

**Table 5:** Demographic and Clinical Baseline Characteristics of the Study Population in Accordance with Cluster Assignment (Total Population)

	Cluster-Related Risk Category			*P* Value
	Low (n = 261)	Intermediate (n = 467)	High (n = 190)	
Age, y	79.5 (76.0-82.7)	80.1 (75.3-82.9)	80.6 (75.8-83.6)	0.412
Men	110 (42.1%)	195 (41.8%)	100 (52.6%)	0.029
BMI, kg/m^2^	25.5 (23.3-28.2)	24.9 (22.3-28.7)	25.4 (22.8-29.1)	0.267
Arterial hypertension	208 (79.7%)	395 (84.6%)	166 (87.4%)	0.073
Diabetes mellitus	52 (19.9%)	131 (28.1%)	58 (30.5%)	0.019
NYHA functional class ≤II	30 (11.5%)	48 (10.3%)	14 (7.37%)	0.342
NYHA functional class III	208 (79.7%)	358 (76.7%)	139 (73.2%)	0.266
NYHA functional class IV	23 (8.81%)	61 (13.1%)	37 (19.5%)	0.004
EuroScore II, %	3.96 (2.36-6.82)	4.87 (3.00-8.43)	5.37 (3.50-9.75)	<0.001
TRI-Score, points	4 (3-6)	6 (4-7)	6 (4-8)	<0.001
eGFR, mL/min	57 (43-72)	53 (34-66)	34 (26-42)	<0.001
NT-proBNP, pg/mL	1,347 (818-1,848)	3,296 (1,810-5,630)	3,639 (1,581-6,941)	<0.001
Hemoglobin, g/dL	12.2 (9.8-13.2)	11.0 (9.1-12.7)	10.8 (9.3-12.4)	<0.001
Bilirubin, mg/dL	0.80 (0.53-1.10)	0.80 (0.57-1.20)	0.92 (0.60-1.20)	0.143
AST, U/L	29 (24-36)	29 (24-36)	28 (23-34)	0.682
ALT, U/L	21 (16-27)	18 (14-25)	16 (13-21)	<0.001
gGT, U/L	83 (44-156)	95 (54-184)	131 (82-237)	<0.001
CAD	101 (38.7%)	201 (43.0%)	84 (44.2%)	0.416
COPD	27 (10.3%)	94 (20.1%)	46 (24.2%)	<0.001
Atrial fibrillation	231 (88.5%)	416 (89.1%)	183 (96.3%)	0.008
Pacemaker	63 (24.1%)	122 (26.1%)	68 (35.8%)	0.016
TR etiology				
Ventricular	131 (50.2%)	253 (54.2%)	119 (62.6%)	0.030
Atrial	105 (40.2%)	168 (36.0%)	52 (27.4%)	0.018
CIED-related	15 (5.75%)	25 (5.35%)	17 (8.95%)	0.209
Primary	10 (3.83%)	21 (4.50%)	2 (1.05%)	0.096

Values are median (IQR) or n (%).

Abbreviations as in Table 1.

**Table 6:** Echocardiographic and Hemodynamic Baseline Characteristics of the Study Population in Accordance With Cluster Assignment (Total Population)

	Cluster-Related Risk Category			*P* Value
	Low (n = 261)	Intermediate (n = 467)	High (n = 190)	
LVEF, %	58 (53-63)	55 (47-61)	55 (47-60)	<0.001
LVESD, mm	33 (27-43)	35 (29-45)	40 (30-50)	<0.001
LVEDD, mm	47 (42-52)	47 (42-52)	49 (44-55)	0.014
LA volume, mL	76 (52-120)	83 (53-115)	86 (46-126)	0.812
sPAP_echocardiography_, mm Hg	35 (29-43)	42 (32-52)	42 (34-53)	<0.001
TAPSE, mm	18 (15-21)	17 (14-19)	17 (14-20)	<0.001
RV FAC, %	41 (35-48)	39 (31-45)	35 (30-42)	<0.001
Basal RV diameter, mm	46 (42-52)	45 (41-50)	49 (44-56)	<0.001
TV EROA, cm^2^	0.53 (0.40-0.80)	0.50 (0.40-0.70)	0.63 (0.45-0.88)	<0.001
TV regurgitation volume, mL	45 (33-63)	44 (35-58)	50 (38-63)	0.072
TR vena contracta width, mm	10 (8-13)	10 (8-12)	12 (9-15)	<0.001
TR ≤ III/V°	128 (49.0%)	267 (57.2%)	80 (42.1%)	0.001
TR = IV/V°	93 (35.6%)	137 (29.3%)	58 (30.5%)	0.206
TR = V/V°	40 (15.3%)	63 (13.5%)	52 (27.4%)	<0.001
RA area, cm^2^	35 (29-45)	31 (27-41)	43 (37-52)	<0.001
Inferior vena cava diameter, mm	23 (20-28)	25 (21-29)	27 (23-32)	<0.001
sPAP_invasive_, mm Hg	37 (32-41)	48 (38-57)	53 (47-60)	<0.001
dPAP, mm Hg	14 (11-17)	20 (16-24)	23 (19-27)	<0.001
mPAP, mm Hg	23 (20-26)	32 (26-37)	36 (32-40)	<0.001
mPCWP, mm Hg	15 (12-17)	21 (16-25)	22 (18-26)	<0.001

Values are median (IQR) or n (%).

Abbreviations as in Table 2.

Conversely, patients from the high-risk category (190 patients) presented with the most pronounced symptomatic burden expressed as NYHA functional class IV (described in 19.5% of patients) and highest NT-proBNP levels (median: 3,639 [IQR: 1,581-6,941] pg/mL). Moreover, they were also marked by highest mPAP levels (median: 36 [IQR: 32-40] mm Hg) and most severely enlarged RA areas (median: 43 [IQR: 37-52] cm^2^). Severe cardiac congestion in patients from the high-risk category was also mirrored in dilated inferior vena cava diameters (median: 27 [IQR: 23-32] mm) and elevated levels of gamma-glutamyl transferase (median: 131 [IQR: 82-237] U/l). Impaired renal function was most critical in this category (median eGFR: 34 [IQR: 26-42] mL/min). The proportion of men was highest in the high-risk category (52.6% vs 42.1% and 41.8% in low-risk and intermediate-risk categories, respectively; *P* = 0.029), and the prevalence of comorbidities such as chronic obstructive pulmonary disease (diagnosed in 24.2%) and atrial fibrillation (diagnosed in 96.3%) also ranged higher than that in low-risk and intermediate-risk categories. Despite the enlargement of the RA, an atrial form of TR was found in only 27.4% of cases, while a ventricular form was diagnosed in 62.6%. With a torrential TR diagnosed in 27.4% of patients (vs 15.3% and 13.5% in low-risk and intermediate-risk categories, respectively; *P* < 0.001), a TR reduction to II° or less was only achieved in 68.4% of cases (vs 81.6% in both low-risk and intermediate-risk categories, respectively; *P* < 0.001) (Table 7). Accordingly, 1-year, 2-year, and 5-year survival rates after TTVI in patients from the high-risk category ranged at only 67.0%, 52.6%, and 18.1%, respectively (Central Illustration, Figure 3).

**Table 7:** Procedural Success Rates (Total Population)

	Cluster-Related Risk Category			*P* Value
	Low (n = 261)	Intermediate (n = 467)	High (n = 190)	
TR reduction by at least 1 grade	242 (92.7%)	429 (91.8%)	172 (90.5%)	0.702
Residual TR ≤II/V°	213 (81.6%)	381 (81.6%)	130 (68.4%)	<0.001

Values are n (%).

In addition, an analysis on a strictly selected sub-population, excluding patients with heterotopic valve implantation, prior TV surgery or TV intervention, and those with concomitant, at least moderate, mitral regurgitation, demonstrated that our model continued to effectively stratify patients into low-risk, intermediate-risk, and high-risk categories (Supplemental Figure 6).

### Comparison with established tools for risk stratification (originally developed in surgically treated patients)

**Figure 4:**
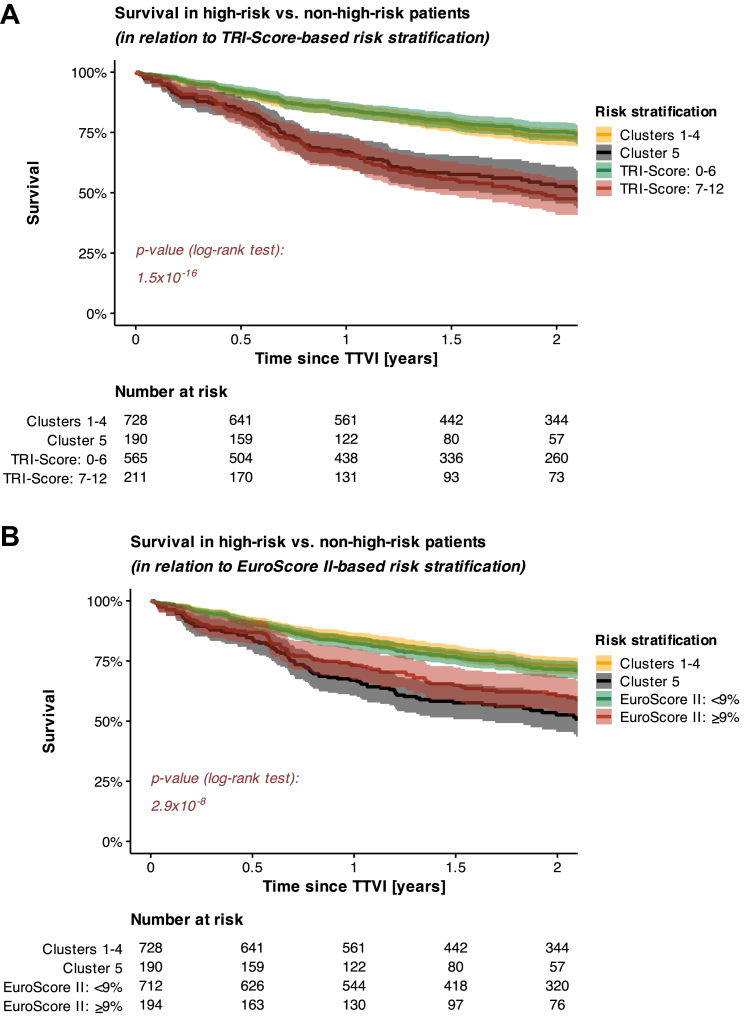
**Survival in High-Risk vs Non-High-Risk Patients (Stratified by Either Survival Tree-Based Modelling or TRI-Score/EuroScore II)** TRI-score (A) assessment was available for 776 out of 918 patients (84.5%), while EuroScore II (B) assessment was available for 906 out of 918 patients (98.7%).

Upon examining the survival outcomes for high-risk patients from Cluster 5, it was further observed that these patients, identified through just 3 steps by our simplified outcome-prediction model, had survival rates comparable to those of patients with a TRI-Score of at least 7 out of 12 points (Figure 4A, Supplemental Figure 7A). This finding carries 2 important implications.

1) It shows that the allocation to Cluster 5 is associated with a risk of mortality similar to that reflected by higher TRI-Score values, suggesting the model’s effectiveness in risk stratification.
2) It underscores the model’s practicality as those high-risk patients were identified by meeting only 3 criteria (mPAP >28 mm Hg, RA area >32.5 cm^2^, and eGFR ≤51 mL/min) (Central Illustration) instead of evaluating 8 criteria as required for the assessment of the TRI-Score.

Furthermore, a re-classification analysis comparing the stratification of patients for 2-year mortality after TTVI by either a TRI-Score ≥7 points or assignment to high-risk Cluster 5 demonstrated no significant differences in risk stratification effectiveness (net re-classification index: −0.04; *P* = 0.407).

On top of that, another re-classification analysis further comparing patient stratification for 2-year mortality after TTVI, using either a EuroScore II ≥ 9% or assignment to high-risk Cluster 5, revealed that our survival tree-based model significantly improves risk-stratification effectiveness (net re-classification index: 0.11; *P* = 0.021) (Figure 4B, Supplemental Figure 7B).

### Assessment of residual TR to update survival prognostication after TTVI

**Figure 5:**
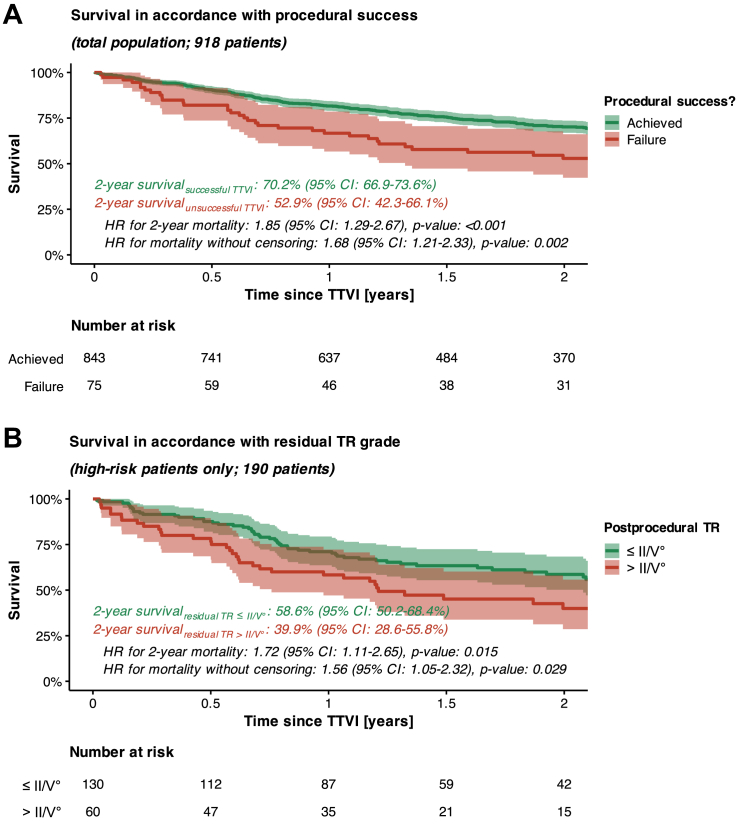
Assessment of Residual TR to Update Risk Stratification After TTVI Survival in accordance with (A) procedural success and (B) residual TR grade.

Procedural success of TTVI was associated with better survival outcome than failure to achieve any reduction in TR severity. Specifically, the absence of any TR reduction after TTVI was associated with a higher 2-year mortality rate (HR: 1.85 [95% CI: 1.29-2.67], *P* < 0.001) (Figure 5A). Irrespective of the definitions of procedural success, our data also show that increasing severity of residual TR translated into higher mortality after TTVI; especially patients with residual TR ≥III/V° at post-procedural echocardiography (a finding detected in 194 out of 918 patients, that is, in 21.1%) experienced significantly higher mortality than those patients with residual TR ≤II/V° (Supplemental Figure 8). Consequently, assessment of residual TR may be used to update survival prognostication after the procedure, an aspect that holds particular relevance for high-risk patients categorized within Cluster 5. In those high-risk patients from Cluster 5 with residual TR ≥III/V°, 2-year survival was only 39.9% (95% CI: 28.6%-55.8%). This is significantly worse than the 2-year survival rate observed in high-risk patients from Cluster 5 with residual TR ≤II/V° (2-year survival: 58.6% [95% CI: 50.2%-68.4%], *P* = 0.015) (Figure 5B).

## Discussion

### On the implications for clinical practice and future research

In this study, we have developed a straightforward, supervised machine learning model to stratify patients with severe TR into distinct risk phenotypes for 2-year mortality following TTVI. Our model holds promising implications for clinical practice and future research. First, this model can guide patient counselling by providing a refined risk-assessment tool, as our survival tree-based model is the first of its kind to be specifically tailored for patients undergoing TTVI. Our model leverages a comprehensive dataset encompassing clinical, laboratory, echocardiographic, and hemodynamic parameters from a large, multicentric registry of patients treated with TTVI. Prior studies primarily focused on patients with severe TR undergoing surgical or conservative treatments;^7^, ^8^, ^9^, ^10^ notably, key predictors of survival in these cohorts—such as pulmonary artery pressure levels, right ventricular function, and renal status—also emerged as important in our survival tree-based model, underscoring their universal prognostic value. However, it was yet important to develop a model specifically for patients undergoing TTVI because TTVI can modify survival prospects as suggested by retrospective studies matching patients undergoing TTVI or receiving conservative treatment only.^11^^,^^12^ Crucially, the success of TTVI—particularly in reducing TR severity—is a vital prognostic factor as evidenced in our study showing that patients with successful TTVI live longer than patients with procedural failure (defined as no reduction in TR severity). For high-risk patients from Cluster 5, we were able to show that persistent TR ≥III/V° was associated with a 2-year survival rate of just 39.9% (95% CI: 28.6%-55.8%). Despite its clinical relevance, however, those data are inherently accessible only after the procedure. Consequently, post-procedural data such as residual TR grade are no viable predictors for a prognostic model intended to guide preprocedural consultations about potential risks, benefits, and anticipated survival outcome. Therefore, the integration of postprocedural data, such as the degree of TR reduction, should be reserved for fine-tuning survival predictions after TTVI.

Another critical application of our survival tree-based model lies in its potential to guide clinical decision-making regarding the timing of TTVI and patient selection. For example, the model identifies progressive enlargement of RA area as a key indicator for poor prognosis. This finding is particularly relevant during the “watch and wait” period in the treatment of severe TR. Progressive RA enlargement, despite maximal dosing of diuretics, signals advancing right-heart failure accompanied by signs of congestion. Such detailed insights provided by the model enable clinicians to make informed decisions about the optimal timing for intervention, thus promising to improve patient outcomes.

Lastly, our model can refine clinical trial designs by facilitating the enrollment of high-risk patients, thereby enriching event data and improving the assessment of treatment effects ascribed to TTVI. This application could become particularly helpful in light of findings from the TRILUMINATE Pivotal trial, the only randomized trial to date. This study demonstrated that TTVI in addition to guideline-directed medical therapy improves the quality of life in an otherwise highly symptomatic patient cohort; however, no differences in rates of all-cause death or hospitalization for heart failure were seen at 1 year after randomization.^13^ Contrastingly, a recent analysis of a real-world multicenter registry encompassing 962 patients revealed that the TRILUMINATE trial’s inclusion criteria were met by only 54.8% of cases who presented with better left ventricular function and fewer comorbidities than ineligible patients.^14^ Importantly, 1-year survival rates differed significantly between eligible and ineligible patients (84.7% vs 74.9%; HR: 1.71 [95% CI: 1.27-2.30]). These findings suggest that TTVI could potentially yield more pronounced benefits in terms of hard clinical endpoints among patients with a more severe illness—a group our model identifies as intermediate-risk (Clusters 2-4) or high-risk (Cluster 5) categories. Therefore, our model's ability to stratify patients by risk category can be instrumental in selecting appropriate candidates for clinical trials, potentially revealing the hoped-for therapeutic potential of TTVI in a broader, more complex patient population.

### Moving from traditional risk scores derived from surgical patients to machine learning models for mortality prediction in TTVI patients

Intriguingly, the TRI-Score, developed to predict in-hospital mortality in patients undergoing tricuspid valve surgery, yielded similar median scores (6 points) for patients in both intermediate-risk and high-risk categories (*P* = 0.140) (Table 5). However, 1-year and 2-year survival differed significantly between these groups. This disparity suggests that the TRI-Score may not effectively discern risk phenotypes among TTVI candidates. Of note, such observations should not be seen as a criticism of the TRI-Score, but rather as a justification for a specific risk-assessment tool for TTVI patients. Our model, based on just 4 key parameters, differs notably from the TRI-Score (which necessitates the evaluation of 8 parameters) by including pulmonary hypertension—a recognized prognostic factor in severe TR cases.^15^, ^16^, ^17^ This inclusion potentially enhances its predictive accuracy for TTVI patient outcomes, while the overall application of our survival tree-based model is simpler and more user-friendly (its intuitive, easy-to-navigate tree structure makes survival prediction accessible without the need of any calculations) than the TRI-Score.

Furthermore, our simplified prediction tool designated to predict survival in TTVI patients significantly outperformed the EuroScore II in identifying high-risk patients for 2-year mortality. This superiority is important given that the EuroScore II was originally developed to estimate perioperative mortality risk in a broad spectrum of cardiac surgeries, including bypass operations, rather than specifically for patients undergoing transcatheter interventions. Notably, while the EuroScore II incorporates female sex as a risk factor, recent studies have demonstrated that survival outcomes for women undergoing TTVI are comparable to those of their male counterparts.^18^^,^^19^ Therefore, EuroScore II-derived predictions, which assign a uniformly poorer prognosis to all women undergoing TTVI for severe TR, warrant cautious interpretation.

### Facilitating pulmonary hypertension assessment with artificial intelligence-enhanced echocardiography

Importantly, the assessment of pulmonary hypertension in our model is based on right-heart catheterization data, a procedure recommended for all patients being considered for TTVI. However, right-heart catheterization is invasive and necessitates specialized infrastructure, limiting its accessibility. Alternatively, echocardiography-derived systolic pulmonary artery pressure (sPAP) levels are more readily obtained but are known to systematically underestimate pulmonary hypertension in patients with severe TR.^16^^,^^17^ To circumvent this issue, our team has developed and refined an extreme gradient boosting algorithm capable of predicting mPAP levels with high accuracy using data from 10 routine transthoracic echocardiography parameters.^17^^,^^20^ This approach promises the diagnostic accuracy of right-heart catheterization and holds the potential to either supplement or partially replace it in certain clinical scenarios. It is especially beneficial for frontline healthcare providers (eg, practicing cardiologists in outpatient settings) without immediate access to right-heart catheterization facilities. Future investigations are warranted to assess the efficacy of our survival tree-based model when incorporating these artificial intelligence–predicted mPAP values in place of those derived from right-heart catheterization; an outlook into the future is presented in Supplemental Figure 9.

### Contextualizing the right atrium in TR pathophysiology

Secondary TR, comprising the majority of TR cases, typically results from annular dilatation and leaflet malcoaptation due to right ventricular remodeling. This remodeling is often a consequence of pulmonary hypertension linked to left heart failure or pulmonary diseases. Interestingly, about 10% to 20% of secondary TR patients exhibit no upstream pathology. Previously termed isolated or idiopathic TR, emerging evidence now suggests that long-standing atrial fibrillation can induce RA enlargement, tricuspid valve annular dilatation, and subsequent TR evolution.^21^ These cases are increasingly recognized as atrial secondary TR.^22^ Given that atrial secondary TR inherently involves less right ventricular dysfunction than ventricular secondary TR, these patients may exhibit more favorable survival outcomes.^23^ This observation is reflected in our study's low-risk phenotypes within Cluster 1, where 40.2% of patients were diagnosed with atrial secondary TR.

Our survival tree-based model’s hierarchical structure mirrors these pathophysiological considerations. It first categorizes patients based on mPAP levels, followed by an evaluation of RA area size if mPAP is elevated. Consequently, high-risk phenotypes in Cluster 5 demonstrate severe pulmonary hypertension (median mPAP: 36 [IQR: 32-40] mm Hg) coupled with significant RA enlargement (median RA area: 43 [IQR: 37-52] cm^2^), markedly exceeding the normal reference value (≤18 cm^2^).^24^ This extensive RA enlargement likely signifies advanced-stage ventricular secondary TR, highlighting the model's capacity to capture complex TR progression dynamics.

### Integrating TAPSE and mPAP for better assessment of right ventricular function in patients with severe TR

Since right ventricular function, as measured by TAPSE, was significantly associated with mortality as shown in the univariate Cox regression analysis (HR per 10-mm increment in TAPSE: 0.95 [95% CI: 0.92-0.97], *P* < 0.001) (Table 4), TAPSE was included among the input parameters for developing the survival tree-based model. However, the unsupervised machine learning algorithm identified mPAP, RA area, NT-proBNP, and eGFR as the most important parameters for stratifying patients into distinct risk categories. Moreover, group-wise comparisons between intermediate- and high-risk patients did not reveal significant differences in TAPSE levels (median TAPSE: 17 [IQR: 14-19] mm in intermediate-risk patients vs 17 [IQR: 14-20] mm in high-risk patients, *P* = 0.677) (Table 6). This finding suggests that focusing solely on right ventricular function may overlook the multifactorial etiology of TR, as well as the complex interplay and the nonsequential nature of co-existing cardiac and extra-cardiac impairments. Therefore, a holistic assessment is crucial for accurate survival prognostication. Notably, differences in right ventricular function between intermediate-risk and high-risk patients became evident, when right ventricular function expressed as TAPSE was put into context to pulmonary artery pressure levels expressed as mPAP (median TAPSE/mPAP ratio: 0.519 [IQR: 0.400-0.667] mm/mm Hg in intermediate-risk patients vs 0.452 [IQR: 0.364-0.562] mm/mm Hg in high-risk patients, *P* < 0.001) (Supplemental Table 2).

### Comparison with conservative treatment to identify cluster-related survival benefits from TTVI

As a risk-stratification model with potential consequences of excluding patients from intervention due to expected futility, it is crucial to have a randomized control group of patients receiving only conservative treatment for comparison. Acknowledging that we cannot provide such gold standard data, we conducted an analysis on patients who were declined for TTVI. Among 83 patients declined for TTVI (mainly due to anatomical criteria) (Supplemental Figure 10A), median survival period was only 2.20 years, and 2-year survival was significantly worse than that in those patients undergoing TTVI (HR for 2-year mortality: 1.55 [95% CI: 1.09-2.21], *P* = 0.015) (Supplemental Figures 10B and 10C). Importantly, clinical baseline characteristics between TTVI and conservative cohorts were similar with regards to age, sex, renal function, and level of decompensation expressed as NT-proBNP (Supplemental Table 3). However, patients declined for TTVI had worse left ventricular function, higher sPAP_echocardiography_ levels, more severely dilated right ventricles, and larger coaptation defects of the tricuspid valve (Supplemental Table 4). Comparing HRs for mortality per cluster-related risk category between TTVI and conservative cohorts revealed that only patients in the intermediate risk category derive a statistically significant survival benefit from TTVI (HR for uncensored mortality: 0.59 [95% CI: 0.37-0.91], *P* = 0.018) (Supplemental Figure 11). These hypothesis-generating data might emphasize the importance of finding the optimal timing for intervention, as too early may not yield a survival-prolonging effect from TTVI, while too late may be insufficient to reverse the cardiac and extra-cardiac damage.

### Study Limitations

Being a retrospective, observational, nonrandomized register study with inherent weaknesses, 3 major limitations of our analysis merit consideration. First, this is a registry study enrolling only patients with severe TR who underwent TTVI; a randomized control group of patients conservatively treated with optimized guideline-directed medical therapy is missing. This study therefore cannot draw any conclusions about the net benefit of TTVI vs conservative treatment per cluster. Second, we acknowledge that this study is based on data which were generated during clinical routine in a real-life scenario, meaning that no central core laboratory was involved. Furthermore, right ventricular function was assessed by echocardiography alone because it is widely available and easily reproducible. However, TAPSE as a parameter for right-ventricular systolic function measures right-ventricular motion only at the basal level, which is particularly problematic when pathological remodeling has already occurred. Future studies should incorporate additional quantitative markers of right ventricular dysfunction, such as right ventricular free wall strain, to further refine our model. On another note, magnetic resonance imaging is the gold standard for assessing right-heart function and structure—particularly useful in patients with both severe TR and right ventricular dysfunction. Incorporating large, raw image data from magnetic resonance imaging could therefore enhance our model’s accuracy and reliability. However, the limited accessibility to magnetic resonance imaging scanners could possibly restrict the clinical applicability of such a future model. Third, a systematic and complete re-assessment of cardiac status and end-organ damage following TTVI is missing for now, but future studies need to shed light on the trajectory towards recovery or further decline especially in high-risk patients from Cluster 5. As shown by our clustering approach, those patients demand closest monitoring. As a minor limitation, we acknowledge that our classification scheme for determining the etiology of TR may be prone to oversimplification. Specifically, the etiology of TR was assessed at the time patients presented with severe TR. However, differentiating the initial etiology of TR in severely ill patients can be challenging, as TR itself can exacerbate right-heart remodeling, leading to a further worsening of the condition. For instance, in patients with initial cardiac implantable electronic device-related TR, long-standing TR will inevitably result in RA enlargement. According to our stepwise classification system, however, these patients would be classified as having secondary atrial TR, which may not accurately reflect the initial pathophysiological mechanisms. A further minor limitation of our study is the reduced sample size in the strictly selected sub-population, which resulted from the exclusion of patients with specific prior interventions and comorbid conditions. This reduction led to broader confidence intervals and overlapping Kaplan-Meier curves among the risk categories shown in Supplemental Figure 6B. While the wider confidence intervals may affect the precision of survival estimates, they do not compromise the model’s ability to distinguish meaningful differences between low-, intermediate-, and high-risk groups. Nonetheless, future studies with larger datasets are warranted to refine these estimates and improve the robustness of survival predictions. Lastly, as TTVI becomes more widespread, future studies with larger populations will enable the development of more complex models with additional decision nodes, potentially enhancing prognostic accuracy and further refining patient stratification.

## Conclusions

This survival tree-based model demonstrates that cardiac and extra-cardiac factors are important for prognosis for 2-year survival after TTVI. The model is simple and has been validated in an external validation cohort. The role of this model in clinical practice requires future study.

Perspectives **COMPETENCY IN PATIENT CARE AND PROCEDURAL SKILLS:** The nature of severe TR is complex, and so is mortality prediction in those patients, especially when considering both cardiac and extra-cardiac factors. The introduction of a machine learning-derived survival tree-based model provides a novel approach to categorizing patients with severe TR into distinct risk phenotypes. This model is the first of its kind to prognosticate survival in patients undergoing TTVI by considering a comprehensive set of laboratory, echocardiographic, and hemodynamic input parameters.

**TRANSLATIONAL OUTLOOK:** Future research should focus on refining this model, particularly by incorporating postprocedural data to reassess patient prognosis. Further studies might also explore the model's utility in guiding clinical decision-making, patient counseling, and enhancing the design of clinical trials, especially for high-risk patient populations.

## Funding Support and Author Disclosures

Dr Fortmeier has received funding from the 10.13039/501100006254Ruhr University Bochum (Female Clinician Scientist Grant). Dr Lachmann has received funding from the 10.13039/501100005713Technical University of Munich (Clinician Scientist Grant), from the German Center for Cardiovascular Research (DZHK; Postdoc Start-up Grant on Advancing Digital Aspects), and from the 10.13039/501100005971German Heart Foundation. Amelie Hesse received funding from the 10.13039/501100010578German Cardiac Society (DGK; Otto Hess Doctoral Scholarship). All other authors have reported that they have no relationships relevant to the contents of this paper to disclose.
