# Supplementary material for: Simplified Outcome Prediction in Patients Undergoing Transcatheter Tricuspid Valve Intervention by Survival Tree-Based Modelling
Source: JACC Adv. 2025 Jan 22;4(2):101575. doi: 10.1016/j.jacadv.2024.101575 (PMC11791227; doi:10.1016/j.jacadv.2024.101575)
Supplement: Supplemental material [file mmc1.docx]

***Supplemental Appendix***

**Methods**

***Extended Description of the Study Population***

For the development of the survival tree-based model, the derivation cohort was assembled from patient data collected at three independent German institutions: the Heart and Diabetes Center North Rhine-Westphalia in Bad Oeynhausen, the Ludwig Maximilians University Hospital in Munich, and the Heart Center at the University of Leipzig. An external validation cohort was also established, comprising patients who received similar treatment at the Heart Center of the University of Cologne Hospital, Germany, and at the Department of Cardiology at Bern University Hospital, Switzerland. This approach ensured a derivation-to-validation split ratio of approximately 67:33, with the derivation cohort comprising 631 patients (68.7% of the total population) and the external validation cohort comprising 287 patients (31.3%). Importantly, patients in the validation cohort were drawn exclusively from hospitals not involved in the model development process, providing a true external validation of the model.

Furthermore, patients with severe TR, who were initially considered for TTVI, but were deemed unsuitable (as assessed by the local heart team), and who were therefore treated with maximally tolerated, optimal medical therapy alone, were recruited from two of the centers mentioned above and hereinafter served as a reference group of patients receiving conservative treatment only.

***Echocardiographic Analysis***

All echocardiographic studies were performed by experienced institutional cardiologists during clinical routine with commercially available equipment (Philips Medical and General Electric systems). Left ventricular ejection fraction was measured using the biplane method. Tricuspid annular plane systolic excursion (TAPSE) was assessed through an apical four-chamber view utilizing the M-mode ultrasound to measure the movement of the tricuspid annulus in the longitudinal direction. Pulmonary hypertension assessment was a routine part of the preprocedural transthoracic echocardiographic examination. The echocardiographic systolic pulmonary artery pressure (sPAP_echocardiography_) was derived by summing the transvalvular gradient across the tricuspid valve (estimated from the continuous wave Doppler profile of the TR jet) with the right atrial (RA) pressure. The latter was inferred from the diameter and collapsibility of the inferior vena cava, as outlined in contemporary guidelines (1,2).

***Right Heart Catheterization***

A 7 French Swan-Ganz catheter was routinely used for preprocedural right heart catheterization via femoral access. Systolic and diastolic pulmonary artery pressure (sPAP and dPAP) levels were directly recorded. Mean pulmonary artery pressure (mPAP) levels were calculated as mPAP = dPAP + 1/3 x (sPAP – dPAP).

***Etiology of TR***

Etiology of TR was delineated following the recent classification proposed by Praz *et al.* (3). This comprehensive scheme distinguishes between primary TR, secondary TR (subdivided into functional atrial and ventricular TR), and TR related to cardiac implantable electronic devices (see the stepwise classification scheme depicted in Supplementary Figure 1 for a detailed understanding).

***Procedural Success Definition***

Procedural success was defined as a device successfully implanted and delivery system retrieved, with TR reduction by at least one grade and/ or a residual TR grade ≤ II/V° as evaluated on transthoracic echocardiography before discharge (that is 2-5 days after the procedure).

***Clinical Endpoint Definition***

As a population of elderly and multimorbid patients was studied, postprocedural 2-year all-cause mortality was defined as a clinically meaningful primary outcome measure. This timeframe offers a balanced perspective: on the one hand, it avoids the pitfalls of shorter follow-up periods, which may risk insufficient event data to discern survival differences; on the other hand, it mitigates the potential for confounding factors more common in extended follow-up durations - such as incidental mortality from unrelated causes.

***TRI-Score Assessment***

The TRI-Score was assessed as described by Dreyfuss et al. (4) for patients without missing data. For those patients lacking any data, the TRI-Score could not be computed, and those patients were excluded from further sub-analyses.

***Artificial Intelligence-enabled mPAP Prediction***

The methodology for mPAP prediction by employing an extreme gradient boosting algorithm using standard echocardiographic parameters as data input has been extensively described elsewhere (5,6). Echocardiographic parameters serving as input variables included left ventricular ejection fraction, left ventricular end-systolic diameter, left atrial area, estimated systolic pulmonary artery pressure (sPAP), basal right ventricular diameter, tricuspid annular plane systolic excursion (TAPSE), TR vena contracta width, tricuspid valve effective regurgitant orifice area, right atrial (RA) area, and inferior vena cava diameter. Missing data were not imputed. Artificial intelligence-derived mPAP values are hereinafter referred to as mPAP_predicted_.

***Statistical Analysis***

Categorical variables are presented as numbers and/or frequencies (%), and continuous variables are given as median and interquartile range (IQR). Chi-square or Fisher’s exact test were used to evaluate the association between categorical variables, and independent-samples Wilcoxon test was used for comparison of continuous variables. To compare more than two groups, Kruskal-Wallis test in combination with pairwise Wilcoxon test with correction for multiple testing (Benjamini-Hochberg method) was used for comparison of continuous variables, as appropriate.

Survival was illustrated using the Kaplan-Meier method, and the log-rank test was applied to compare survival rates. Moreover, a Cox proportional hazards model was used to estimate hazard ratios (HR).

Before developing the survival tree-based model through supervised recursive partitioning within the derivation cohort, a univariate Cox regression analysis on a complementary set of clinical, laboratory, echocardiographic, and hemodynamic variables was performed to identify factors related to all-cause mortality. Variables with a *p*-value ≤ 0.05 in univariate testing were subsequently considered as input parameters for the survival tree-based model.

To address missing data within the selected input parameters for the survival tree-based model, a well-established random forest algorithm was employed to impute values under the assumption of missing at random (7). This step was crucial to ensure that the dataset for training of the model was complete. Importantly, those imputed values were exclusively used for model development; in subsequent analyses such as the comparison of baseline characteristics, we strictly used available, non-imputed data to ensure the integrity and accuracy of these comparisons.

Following imputation, the construction of the survival tree-based model was performed through 5-fold cross-validation using the “partykit“ R package. Each fold of the data was used once as a validation while the k-1 remaining folds formed the training set. Furthermore, we restricted the survival tree-based model to a maximum of three decision nodes to prevent overfitting and to ensure sufficient sample sizes within each cluster. This decision reflects the constraints imposed by the dataset size and prioritizes model simplicity and interpretability over excessive complexity, while also balancing model performance and practical applicability in a clinical setting. Additionally, aligning with this restriction of model complexity in terms of maximum three decision nodes depth for patient-to-cluster assignment, the model’s candidate input parameters from the univariate analysis were further refined to include only the most prognostically relevant variables.

The fitted survival tree-based model was hereinafter applied to patient data from the external validation cohort, and 1-year and 2-year survival rates per cluster were then compared between derivation and validation cohorts to test the predictive validity of the model.

A *p*-value ≤ 0.05 was considered to indicate statistical significance.

All statistical analyses were performed using R statistical software (R version 4.3.2; R Foundation for Statistical Computing, Vienna, Austria; see Supplementary Table 1 for a complete list of employed R packages).

**References**

1. Rudski LG., Lai WW., Afilalo J., et al. Guidelines for the Echocardiographic Assessment of the Right Heart in Adults: A Report from the American Society of Echocardiography. J Am Soc Echocardiogr 2010;23(7):685–713. Doi: 10.1016/j.echo.2010.05.010.

2. Lang RM., Badano LP., Mor-Avi V., et al. Recommendations for Cardiac Chamber Quantification by Echocardiography in Adults: An Update from the American Society of Echocardiography and the European Association of Cardiovascular Imaging. J Am Soc Echocardiogr 2015;28(1):1-39.e14. Doi: 10.1016/j.echo.2014.10.003.

3. Praz F., Muraru D., Kreidel F., et al. Transcatheter treatment for tricuspid valve disease. EuroIntervention 2021;17(10):791–808. Doi: 10.4244/EIJ-D-21-00695.

4. Dreyfus J., Audureau E., Bohbot Y., et al. TRI-SCORE: a new risk score for in-hospital mortality prediction after isolated tricuspid valve surgery. Eur Heart J 2022;43(7):654–62. Doi: 10.1093/eurheartj/ehab679.

5. Fortmeier V., Lachmann M., Körber MI., et al. Solving the Pulmonary Hypertension Paradox in Patients With Severe Tricuspid Regurgitation by Employing Artificial Intelligence. JACC Cardiovasc Interv 2022;15(4):381–94. Doi: 10.1016/j.jcin.2021.12.043.

6. Fortmeier V., Lachmann M., Stolz L., et al. Artificial intelligence–enabled assessment of right ventricular to pulmonary artery coupling in patients undergoing transcatheter tricuspid valve intervention. Eur Heart J - Cardiovasc Imaging 2023:jead324. Doi: 10.1093/ehjci/jead324.

7. Stekhoven DJ., Buhlmann P. MissForest--non-parametric missing value imputation for mixed-type data. Bioinformatics 2012;28(1):112–8. Doi: 10.1093/bioinformatics/btr597.

**Supplemental Table 1: R packages employed in this study.**

| *Amelia*  *caret*  *dplyr*  *forcats*  *foreach*  *gcookbook*  *ggalluvial*  *ggbeeswarm*  *ggExtra*  *ggplot2*  *ggpubr*  *ggrepel*  *ggridges* | *iterators*  *itertools*  *lattice*  *libcoin*  *lubridate*  *missForest*  *mlbench*  *mvtnorm*  *partykit*  *purr*  *randomForest*  *rcompanion*  *Rcpp* | *readr*  *readxl*  *reshape2*  *stringr*  *survival*  *survminer*  *tibble*  *tidyr*  *tidyverse*  *viridis*  *viridisLite*  *xgboost* |
| --- | --- | --- |

**Supplemental Table 2: Comparison of right ventricular to pulmonary artery coupling indices (total population).**

|  | **Cluster-related risk category** | | |  |
| --- | --- | --- | --- | --- |
|  | **Low**  **(*n* = 261)** | **Intermediate**  **(*n* = 467)** | **High**  **(*n* = 190)** | ***p*-value** |
| **TAPSE/sPAP_echocardiography_, median (IQR), mm/mmHg** | 0.507 (0.387-0.667) | 0.405 (0.300-0.529) | 0.395 (0.304-0.520) | <0.001 |
| **TAPSE/mPAP, median (IQR), mm/mmHg** | 0.804 (0.653-0.963) | 0.519 (0.400-0.667) | 0.452 (0.364-0.562) | <0.001 |

mPAP: mean pulmonary artery pressure (as assessed by right heart catheterization); sPAP_echocardiography_: systolic pulmonary artery pressure (as assessed by echocardiography); TAPSE: tricuspid annular plane systolic excursion.

**Supplemental Table 3: Demographic and clinical baseline characteristics of the extended study population.**

|  | |  | | **Treatment** | | | |  |
| --- | --- | --- | --- | --- | --- | --- | --- | --- |
|  | | **All patients**  **(*n* = 1,001)** | | **TTVI**  **(*n* = 918)** | | **Conservative**  **(*n* = 83)** | | ***p*-value** |
| **Age, median (IQR), years** | 80.0 (75.7-83.1) | | 80.0 (75.7-83.1) | | 80.6 (75.0-83.6) | | 0.836 | |
| **Men, No. (%)** | 447 (44.7%) | | 405 (44.1%) | | 42 (50.6%) | | 0.306 | |
| **BMI, median (IQR), kg/m^2^** | 25.3 (22.7-28.7) | | 25.3 (22.7-28.7) | | 25.6 (22.9-28.7) | | 0.338 | |
| **Diabetes mellitus, No. (%)** | 254 (25.3%) | | 241 (26.3%) | | 13 (15.7%) | | 0.046 | |
| **NYHA class ≤ II, No. (%)** | 101 (10.1%) | | 92 (10.0%) | | 9 (10.8%) | | 0.962 | |
| **NYHA class III, No. (%)** | 771 (77.0%) | | 705 (76.8%) | | 66 (79.5%) | | 0.669 | |
| **NYHA class IV, No. (%)** | 129 (12.9%) | | 121 (13.2%) | | 8 (9.64%) | | 0.453 | |
| **EuroScore II, median (IQR), %** | 4.83 (3.03-8.30) | | 4.80 (2.96-8.24) | | 5.91 (4.10-10.7) | | 0.005 | |
| **eGFR, median (IQR), mL/min** | 47 (33-64) | | 47 (33-64) | | 44 (34-56) | | 0.388 | |
| **NT-proBNP, median (IQR), pg/mL** | 2,351 (1,336-4,701) | | 2,304 (1,336-4,634) | | 2,710 (1,335-5,415) | | 0.445 | |
| **CAD, No. (%)** | 426 (42.6%) | | 386 (42.0%) | | 40 (48.2%) | | 0.333 | |
| **COPD, No. (%)** | 177 (17.7%) | | 167 (18.2%) | | 10 (12.0%) | | 0.211 | |
| **Atrial fibrillation, No. (%)** | 903 (90.2%) | | 830 (90.4%) | | 73 (88.0%) | | 0.596 | |
| **Pacemaker, No. (%)** | 281 (28.1%) | | 253 (27.6%) | | 28 (33.7%) | | 0.284 | |

BMI: body mass index; CAD: coronary artery disease; COPD: chronic obstructive pulmonary disease; eGFR: estimated glomerular filtration rate; NYHA: New York Heart Association.

**Supplemental Table 4: Echocardiographic and hemodynamic baseline characteristics of the extended study population.**

|  | |  | **Treatment** | | |  |
| --- | --- | --- | --- | --- | --- | --- |
|  | | **All patients**  **(*n* = 1,001)** | **TTVI**  **(*n* = 918)** | | **Conservative**  **(*n* = 83)** | ***p*-value** |
| **LVEF, median (IQR), %** | 55 (48-61) | | | 55 (49-61) | 54 (45-59) | 0.003 |
| **LVESD, median (IQR), mm** | 34 (28-45) | | | 35 (29-46) | 33 (28-38) | 0.009 |
| **LVEDD, median (IQR), mm** | 47 (42-53) | | | 47 (42-53) | 48 (42-53) | 0.645 |
| **LA volume, median (IQR), mL** | 85 (52-119) | | | 83 (49-118) | 105 (89-148) | 0.002 |
| **sPAP_echocardiography_, median (IQR), mmHg** | 40 (31-50) | | | 40 (31-50) | 47 (36-59) | 0.009 |
| **TAPSE, median (IQR), mm** | 17 (14-20) | | | 17 (14-20) | 16 (13-19) | 0.146 |
| **RV FAC, median (IQR), %** | 39 (32-46) | | | 39 (32-46) | 41 (33-48) | 0.162 |
| **Basal RV diameter, median (IQR), mm** | 47 (42-53) | | | 46 (42-52) | 52 (47-58) | <0.001 |
| **TV EROA, median (IQR), cm^2^** | 0.54 (0.40-0.79) | | | 0.54 (0.40-0.78) | 0.60 (0.30-1.20) | 0.700 |
| **TV regurgitation volume, median (IQR), mL** | 45 (35-61) | | | 45 (35-61) | 44 (30-61) | 0.431 |
| **TR vena contracta width, median (IQR), mm** | 10 (8-14) | | | 10 (8-14) | 12 (9-17) | 0.001 |
| **TR ≤ III/V°, No. (%)** | 529 (52.8%) | | | 475 (51.7%) | 54 (65.1) | 0.027 |
| **TR = IV/V°, No. (%)** | 297 (29.7%) | | | 288 (31.4%) | 9 (10.8%) | <0.001 |
| **TR = V/V°, No. (%)** | 175 (17.5%) | | | 155 (16.9%) | 20 (24.1%) | 0.132 |
| **RA area, median (IQR), cm^2^** | 36 (29-45) | | | 36 (29-45) | 35 (29-45) | 0.819 |
| **Inferior vena cava diameter, median (IQR), mm** | 25 (21-29) | | | 25 (21-29) | 26 (23-31) | 0.042 |
| **mPAP_predicted_, median (IQR), mmHg** | 29 (27-32) | | | 29 (27-32) | 30 (28-33) | 0.020 |

Basal RV diameter: basal right ventricular diameter; LA volume: left atrial volume; LVEDD: left ventricular end-diastolic diameter; LVEF: left ventricular ejection fraction; LVESD: left ventricular end-systolic diameter; mPAP: mean pulmonary artery pressure (predicted); RA area: right atrial area; RV FAC: right ventricular fractional area change; sPAP_echocardiography_: systolic pulmonary artery pressure (as assessed by echocardiography); TAPSE: tricuspid annular plane systolic excursion; TR: tricuspid regurgitation; TR vena contracta width: tricuspid regurgitation vena contracta width; TV regurgitation volume: tricuspid valve regurgitation volume; TV EROA: tricuspid valve effective regurgitant orifice area.

**Supplemental Figure 1: Stepwise classification scheme to define TR etiology.**

CIED: cardiac implantable electronic device; RA: right atrial; RV: right ventricular; TR: tricuspid regurgitation; TV: tricuspid valve.

**Supplemental Figure 2: Extended information on patient enrollment, follow-up, and survival.**

A) Patient enrollment between 2016 and 2022 (blue bars indicate the annual number of patients treated by TTVI; the red line shows the cumulative number of patients being enrolled over the years [918 in total]).

B) Density plot showing time to censoring (survivors) and time to death (non-survivors) in consecutively enrolled patients.

C) Kaplan-Meier survival plot for the entire study population (follow-up beyond the primary study endpoint of 2 years after TTVI).

**Supplemental Figure 3: Imputation of missing values (derivation cohort).**

A) Illustration of missing and present values.

B) Bar plot showing the proportion of missing values per variable.

**Supplemental Figure 4: Hazard ratio for 1-year and 2-year mortality per cluster (comparing derivation and validation cohorts).**

**Supplemental Figure 5: 5-year survival rates in accordance with survival tree-based modelling (total population).**

**Supplemental Figure 6: Risk stratification using survival tree-based modeling in a strictly selected sub-population.**

A) Flow chart with exclusion criteria to eventually homogenize the patient sub-population.

B) Kaplan-Meier survival plot for the strictly selected sub-population.

MR: mitral regurgitation; TTVI: transcatheter tricuspid valve intervention; TV: tricuspid valve.

**Supplemental Figure 7: Survival in high-risk vs. non-high-risk patients (stratified by either survival tree-based modelling or TRI-Score/ EuroScore II; restricted to 5-year outcomes).**

**Supplemental Figure 8: Hazard ratio for mortality per residual TR severity as assessed at post-procedural echocardiography.**

**Supplemental Figure 9: Artificial intelligence-enabled mPAP prediction using routine echocardiography data, and subsequent risk stratification by survival tree-based modeling.**

A) Illustrative example on how to predict mPAP levels using routine echocardiography data. The echocardiographic parameters that were selected as input variables included left ventricular ejection fraction (LVEF), left ventricular end-systolic diameter, left atrial area, estimated systolic pulmonary artery pressure (sPAP_echocardiography_), basal right ventricular diameter, tricuspid annular plane systolic excursion (TAPSE), tricuspid regurgitation (TR) vena contracta width, tricuspid valve effective regurgitant orifice area (TV EROA), right atrial area, and inferior vena cava diameter. For the prediction of mPAP levels, we employed an extreme gradient boosting (XGB) algorithm, as detailed in previous works (*Fortmeier V, et al., Solving the Pulmonary Hypertension Paradox in Patients With Severe Tricuspid Regurgitation by Employing Artificial Intelligence. JACC Cardiovasc Interv. 2022; Fortmeier V, et al., Artificial intelligence–enabled assessment of right ventricular to pulmonary artery coupling in patients undergoing transcatheter tricuspid valve intervention. Eur Heart J - Cardiovasc Imaging. 2023*).

B) Correlation plot (*R* = correlation coefficient by Pearson) showing invasively measured and predicted mPAP levels. Blue line: linear regression line. Gray area: 95% confidence interval.

C) Survival tree-based model to stratify patients into various risk categories. Please note that this stratification model uses predicted mPAP values instead of invasively measured mPAP values.

D) Kaplan-Meier survival plot based on patient-to-cluster assignment using predicted mPAP levels.

**Supplemental Figure 10: Characteristics and survival rates among screen failure patients evaluated for TTVI.**

A) Pie charts illustrating the reasons why patients did not undergo TTVI and were hence treated with conservative therapy only.

B) Kaplan-Meier survival plot showing survival rates among screen failure patients evaluated for TTVI (hereinafter referred to as conservative cohort).

C: Kaplan-Meier survival plot comparing survival rates between patients undergoing TTVI and patients under conservative treatment only.

CTEPH: chronic thromboembolic pulmonary hypertension; HCM: hypertrophic cardiomyopathy; PAH: pulmonary arterial hypertension; TTVI: transcatheter tricuspid valve intervention.

**Supplemental Figure 11: Hazard ratios for mortality in accordance with cluster-related risk category and treatment (TTVI vs. conservative therapy alone).**

To assign patients to clusters, mPAP levels were imputed by an extreme gradient boosting algorithm using echocardiography input data as outlined in the methods section and in Supplementary Figure 9.
